# Lacking Evidence to Recommend Neoadjuvant Chemotherapy and Definitive Radiotherapy in Muscle-Invasive Bladder Cancer: In response to: Trimodality Therapy for Muscle-Invasive Bladder Cancer: Recent Advances and Unanswered Questions published by Di Maria Jiang et al. Current Oncology Reports 2020, https://doi.org/10.1007/s11912-020-0880-5

**DOI:** 10.1007/s11912-020-01012-8

**Published:** 2021-01-20

**Authors:** Dirk Böhmer, Arne Grün

**Affiliations:** 1 Department of Radiation Oncology, Charité University Medicine, Campus Benjamin Franklin, Hindenburgdamm 30, 12203 Berlin, Germany; 2 Department of Radiation Oncology, Charité University Medicine, Campus Virchow Klinikum, Berlin, Germany

**Keywords:** Bladder cancer, Trimodality treatment, Neoadjuvant chemotherapy

## Abstract

We comment on the paper of Di Maria Jiang et al. published in Current Oncology Reports 2020, 10.1007/s11912-020-0880-5. We disagree on a major recommendation of the authors because of lacking evidence. This response is considered to be important for readers of Current Oncology Reports.

Letter to the editor:

We read with great interest the article by Jiang et al. on their review regarding trimodality therapy in advanced muscle-invasive bladder cancer (MIBC). They comprehensively address issues on bladder preservation strategies from the past to present. They present an elaborate view into the future with modern immunotherapy concepts and possible genetic pathway targets. The authors state that neoadjuvant chemotherapy (NAC) is the preferred setting for all patients regardless of whether patients receive radiotherapy or cystectomy.

We strongly disagree with the authors’ interpretation of results regarding neoadjuvant chemotherapy and subsequent chemoradiation for the following reasons.

Only one randomized trial is available with NAC involving patients who are eligible for cystectomy or chemoradiation. Patients in the BA06 30894 trial were randomized to either neoadjuvant chemotherapy or no chemotherapy. The type of definitive treatment was not randomized but the choice of the patient and/or physician. Although the numbers of randomized patients receiving radiotherapy (RT) (*N* = 403) and cystectomy (*N* = 428) seem almost evenly distributed the BA06-authors clearly point out that several factors, namely WHO performance status, age, number of T2 tumors, and number of N0 patients, respectively, were unbalanced among both groups.

Results show a significant reduction in the risk of death for the cystectomy group (HR: 0.74; *p* = 0.022). Risk reduction for the radiotherapy group is in fact 20% (HR: 0.80) but this difference is not statistically significant (*p* = 0.070).

Locoregional disease-free survival is improved again by 26% for the cystectomy group (*p* = 0.019) but only by 9% for the RT group with *p* = 0.417 [1].

The second phase III trial, RTOG 89-03, evaluated neoadjuvant chemotherapy before trimodality treatment. Due to a high rate of chemotherapy related toxicities, it closed prematurely. With 123 patients randomized neither overall survival nor metastases-free survival were significantly different [2]. The remaining studies presented by the authors are either phase II trials, cohort-studies, or ongoing studies without published results. Furthermore, the authors do not add specific evidence added to support their assumption.

Thus, it is our belief that the conclusion to prefer neoadjuvant chemotherapy in patients who undergo bladder-sparing approaches combined with radiotherapy is not supported by any high-level evidence.

The authors should acknowledge that neoadjuvant chemotherapy is not considered standard in patients undergoing trimodality treatment for muscle-invasive bladder cancer.
